# Contrasting the Effects of Aspartic Acid and Glycine in Free Amino Acid and Peptide Forms on the Growth Rate, Morphology, Composition, and Structure of Synthetic Aragonites

**DOI:** 10.1021/acs.cgd.4c00766

**Published:** 2024-11-03

**Authors:** Giacomo Gardella, Maria Cristina Castillo Alvarez, Sam Presslee, Adrian A. Finch, Kirsty Penkman, Roland Kröger, Matthieu Clog, Nicola Allison

**Affiliations:** † School of Earth and Environmental Sciences, University of St. Andrews, St Andrews KY16 9TS, U.K.; ‡ Scottish Oceans Institute, University of St. Andrews, St Andrews KY16 8LB, U.K.; § Department of Chemistry, University of York, York YO10 5DD, U.K.; ∥ Department of Physics, University of York, York YO10 5DD, U.K.; ⊥ SUERC, University of Glasgow, Glasgow G75 0QF, U.K.

## Abstract

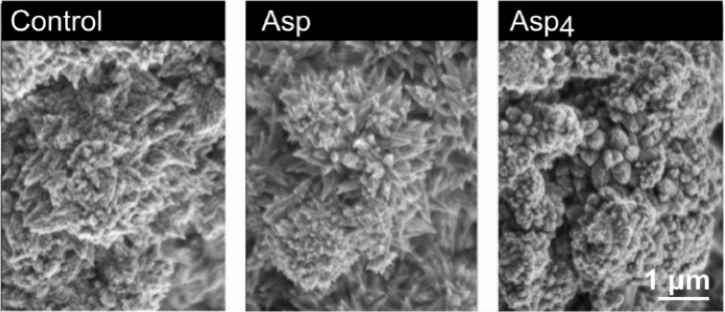

Corals and mollusks produce aragonite skeletons and shells containing highly acidic proteins, rich in aspartic acid (Asp) and glycine (Gly). These biomolecules are pivotal in controlling biomineral formation. We explore the effects of l-Asp, Gly, and two peptides: glycyl-l-aspartic acid (Gly-Asp) and tetra-aspartic acid (Asp_4_) on the precipitation rate, crystal morphology, and CO_3_ group rotational disorder (inferred from Raman spectroscopy) in aragonite precipitated in vitro at the approximate pH, [Ca^2+^], and Ω_ar_ occurring in coral calcification media. All of the biomolecules, except Gly, inhibit aragonite precipitation. Biomolecules are incorporated into the aragonite and create CO_3_ group rotational disorder in the following order: Asp_4_ > Asp = Gly-Asp > Gly. Asp_4_ inhibits aragonite precipitation more than Asp at comparable solution concentrations, but Asp reduces aragonite precipitation more effectively than Asp_4_ for each Asp residue incorporated into the aragonite. At the highest solution concentration, the molar ratio of Asp_4_:CaCO_3_ in the aragonite is 1:690. We observe a significant inverse relationship between the aragonite precipitation rate and aragonite Raman spectrum ν_1_ peak fwhm across the entire data set. Tetra-aspartic acid inhibits aragonite precipitation at all concentrations, suggesting that the aspartic acid-rich domains of coral skeletal proteins influence biomineralization by suppressing mineral formation, thereby shaping skeletal morphology and preventing uncontrolled precipitation.

## Abstract 2

Corals and mollusks produce aragonite containing proteins rich in aspartic acid and glycine. We precipitated aragonite from modified seawater, at the pH and Ω_ar_ believed to occur in coral calcification fluids and in the presence and absence of free amino acids and peptides containing aspartic acid and glycine. We explore biomolecule effects on the precipitation rate and aragonite morphology and structure.

## 1 Introduction

Calcareous organisms produce CaCO_3_ structures, which confer protection to the organisms, contribute to habitats, and influence the global carbon cycle. CaCO_3_ biominerals are hierarchically structured, organic–inorganic composite minerals containing a variety of biomolecules.^1,2^ Organisms secrete biomolecules to exert biological control over different stages of biomineralization, including nucleation, growth, and crystal morphology.^2,3^ In addition, biomolecules confer different physical properties on biominerals compared to their fully inorganic counterparts.^4,5^ This allows organisms to improve the mechanical properties (e.g., fracture resistance and hardness) of the biominerals compared to inorganic analogues.^6^

Identifying how biomolecules influence CaCO_3_ formation is critical to understanding their role in biomineralization. CaCO_3_ growth proceeds by the attachment of ions to the crystal surface, with a high probability of attachment at kink sites (disjoints on the crystal surface).^7^ Growth may also reflect the attachment of nanocrystals^8^ or amorphous calcium carbonates (ACCs) as reported in coral aragonite biomineralization.^9^ Biomolecules/additives may affect CaCO_3_ formation by binding the aqueous ions required for mineral formation, thereby altering their availability for precipitation, e.g., amino acids can complex Ca^2+^ in seawater.^10^ Additives may also block attachment sites on the existing crystal surface^7^ and alter the formation of CaCO_3_ precursor phases.^11−13^

Amino acids can influence the formation and stability of CaCO_3_ precursors^14^ and alter the polymorph, crystal shape, and size of precipitated CaCO_3_.^15^ Amino acids and peptides promote or inhibit CaCO_3_ precipitation.^16−18^ In the marine environment, multiple organisms produce aragonite, including polychaete worms,^19^ some foraminifera,^20^ mollusks,^21^ and corals.^22^ Despite this importance, relatively few studies have investigated the influence of biomolecules on aragonite crystallization.^18,23,24^ Identifying the biomolecule role in aragonite formation is especially important as the total organic and amino acid concentration of tropical coral skeletons is increased in specimens cultured under future ocean acidification scenarios.^24−26^ Resolving how biomolecules affect aragonite formation is important in predicting the future accretion rates and structural resilience of coral reefs.

**Figure 1:**
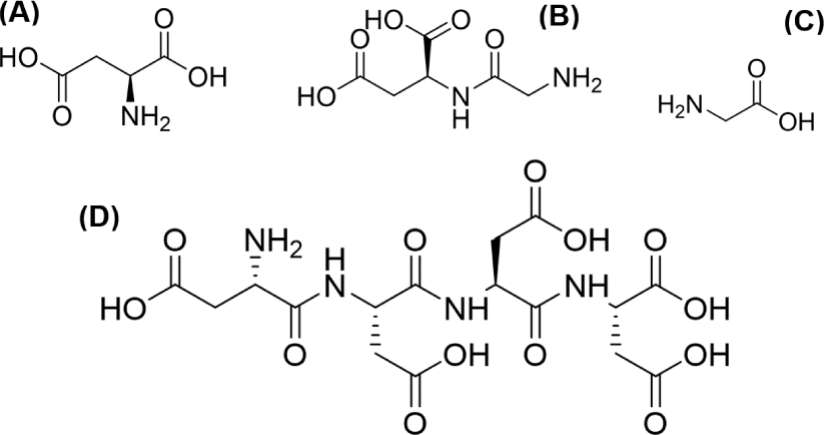
Biomolecules used in this study (A) Asp, (B) Gly-Asp, (C) Gly, and (D) Asp_4_.

In this study, we explore the effects of two free amino acids and two peptides on the precipitation rate, morphology, and CO_3_ group disorder in the crystal lattice of aragonite. Aspartic acid (termed Asp) is an important component of biomineralization proteins in mollusk shells^27−29^ and coral skeletons.^1,24,30^ Glycine (termed Gly) also occurs in high concentrations in coral skeletons^1,24^ and mollusk nacre.^28^ Asp is an acidic amino acid with a COOH side chain, which is deprotonated (to COO^–^) at physiological pH to produce the anion aspartate. Gly is a neutral amino acid, zwitterionic, but overall uncharged at physiological pH.^31^ Both amino acids complex Ca^2+^ in solution^10,32^ and adsorb to calcite crystal faces.^17^ We also explore the effects of the dipeptide glycyl-l-aspartic acid (termed Gly-Asp) and the tetra peptide tetra-aspartic acid (termed Asp_4_). Aspartic acid-rich domains are common in biomineralization proteins^30^ and contrasting the effects of peptides and free amino acids provides information on the role of molecule size in interactions with CaCO_3_ (Figure 1).

For this study, we precipitated aragonite in vitro from modified seawater at the pH and saturation state (Ω_ar_) believed to occur at the calcification site of tropical corals. We used an apparatus designed to maintain the pH and content of the solution at a constant composition.^18^ We determine aragonite precipitation rates in the presence and absence of biomolecules, measure biomolecule incorporation in aragonite by reverse phase high-performance liquid chromatography (RP-HPLC), and explore how biomolecules alter aragonite morphology. Finally, we analyze the precipitates by Raman spectroscopy. The most prominent band in the aragonite Raman spectrum, the ν_1_ band, reflects symmetric C–O stretching in the planar CO_3_ group. An increase in its full width at half-maximum (fwhm, a metric of the peak breadth normalized for peak height) is linked to increased local disorder around this group in the CaCO_3_ lattice.^33,34^ We therefore explore how aragonite formation in the presence of biomolecules affects this disorder.

## 2 Methods

### 2.1 Aragonite Precipitation Experiments

Synthetic aragonites were precipitated using the constant composition technique^35^ using a Metrohm Titrando 902 pH stat titrator. This method relies on the addition of equal volumes of CaCl_2_ and Na_2_CO_3_ to maintain constant pH and Ω, while CaCO_3_ precipitates. For further details of the apparatus used here, see refs^18,23^. The titrants used were 0.4455 M CaCl_2_ + 0.0045 M SrCl_2_ and 0.45 M Na_2_CO_3_. Sr was replenished in the reaction vessel as it substitutes for Ca^2+^ ions in aragonite.^36^ Titrants were added automatically, when the pH of the solution, which decreases due to the precipitation of calcium carbonate, fell below 0.003 pH units of the set value. The standard deviation of pH measurements measured every 1 to 5 s during each titration was <0.005 pH units, and we considered the pH of the solution to be essentially constant. An aragonite seed was added to provide a surface for aragonite growth. For all experiments reported here, aragonite was precipitated from artificial seawater (to the composition of Millero^37^ and with S = 35), filtered with a 0.2 μm polycarbonate filter, and stored in 100 L blacked-out HDPE tanks. Prior to use, the seawater was bubbled with atmospheric air sourced from outside the building (∼416 ppm of *p*CO_2_) and then adjusted to pH_NBS_ = 8.445 and dissolved inorganic carbon concentration ([DIC]) = 4000 μmol kg^–1^ by the addition of 0.6 M Na_2_CO_3_ (to increase DIC) and 2 M HCl or NaOH (to control pH). We estimate solution Ω_aragonite_ = 11.4 using CO2SYS v2.1^38^ with the equilibrium constants for carbonic acid^39^ and KHSO_4_^40^ and using [B]_seawater_.^41^

Where used, biomolecules were dissolved in aliquots of this manipulated seawater and added to the reaction vessel shortly before the start of the experiment, allowing time for final pH adjustments. All biomolecules were sourced from Sigma-Aldrich and had a purity of >97% for Asp_4_ and >99% for all others. pH was monitored using a combined pH electrode and temperature sensor (Metrohm Aquatrode PT1000). The apparatus was cleaned with 0.1 M HCl, and the titrant dosing tubes were submerged in 1 M HCl between experiments.

In the first series of experiments, reactions were conducted with either a) no biomolecule (control), b) 2 mM l-Asp, c) 2 mM Gly, d) 2 mM Gly-Asp dipeptide, or e) 2 mM Asp + 2 mM Gly (both amino acids added separately). Precipitations were conducted in 330 mL of seawater contained in a high density polyethylene (HDPE) beaker maintained in a water bath at 25 °C. The beaker was capped with an ethylene tetrafluoroethylene lid with ports to insert the following: a pH/temperature sensor, a propeller stirrer, a gas tube (supplying air with ∼416 ppm *p*CO_2_ into the headspace), and the two titrant dosing tubes. For each experiment, the seed consisted of 200 ± 2 mg of a *Porites lutea* skeleton, which was wet ground using an agate ball mill to produce a powder with a surface area of 4.70 ± 0.14 m^2^ g^–1^ (mean ± 1 standard deviation, *n* = 3) as determined by the Brunauer–Emmett–Teller technique.^42^ The seed was suspended in 1 mL of the seawater solution, agitated on a vortex mixer, and added to the reaction vessel at the start of the experiment. The experiment proceeded until 7 mL of each titrant was dosed, resulting in the precipitation of ∼315 mg of aragonite in vitro. 5–7 replicates of each treatment were conducted. Over the course of the precipitation, solution temperature varied by <0.5 °C and is considered essentially constant.

The [DIC] of the reaction solution was measured before the seed was added and just before the end of the experiment using an Apollo Sci Tech AS-C3 DIC^43^ for a subset (*n* = 24) of the precipitation experiments. For this, 12 mL of the experiment solution was filtered through a 0.22 μm polyether sulfone syringe filter, and 0.6 mL was injected into the analyzer. The measurement was replicated 5 times after flushing with the filtrate. The DIC analyzer was calibrated before every session with a seawater certified reference material (Dickson batch 171). The difference between measured [DIC] and expected [DIC] was <50 μmol kg^–1^ for controls and <65 μmol kg^–1^ for all other experiments, and the difference between [DIC] at the start and end of the precipitation was 4% on average and always <8%. These minor variations in [DIC] indicate that CO_2_ invasion or outgassing during the experiments was minimal (see the Method section in the Supporting Information). The average change in [DIC] of 4% over a titration is equivalent to a change in Ω_aragonite_ of 0.4.

In the second set of experiments, aragonite was precipitated in the presence of varying concentrations (1–4000 μM) of Asp and Asp_4_. Due to the limited availability of the peptide, these precipitations were conducted in a smaller HDPE beaker filled with 33 mL of artificial seawater. No lid was used, and the pH sensor and titrant tubes were inserted through the top of the beaker. The seed was 20 mg of synthetic aragonite precipitated at Ω_aragonite_ = 11 using the same pH stat titrator utilized in this study and wet ground to yield a surface area of 5.13 m^2^ g^–1^ as determined by the Brunauer–Emmett–Teller technique.^39^ 0.7 mL of each titrant was added to the solution, resulting in the precipitation of ∼32 mg of aragonite. The reaction vessel was kept in a controlled temperature room maintained at 25 °C and was stirred using an 8 mm length magnetic stirrer on a stirrer plate. Over the course of the precipitation, the solution temperature varied by up to 1 °C. The reaction vessel was too small to permit DIC analyses at the start and end of precipitations, but solutions set up using the same method had [DIC] of 3950–4100 μmol kg^–1^. 2–3 replicates were conducted for each treatment, with the exception of the experiment with 1000 μM Asp_4_, which was only conducted once, as this final experiment took more than 24 h.

For both series of experiments, the solids (original seed plus precipitate) were recovered by filtering the reaction solution through a 0.2 μm polycarbonate filter (Nucleopore), rinsing with deionized water (18.2 Megaohm) and ethanol, drying at room temperature, and then storing in a desiccator.

**Figure 2:**
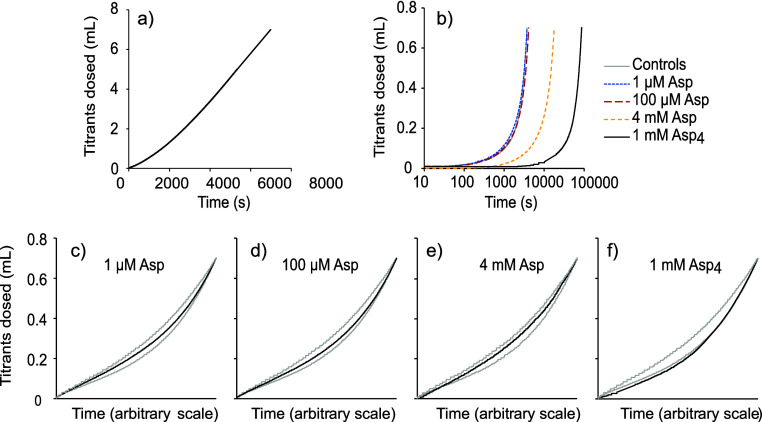
Profiles showing titrant dosing as a function of time in a) a control experiment in 330 mL of solution and b) control, Asp, and Asp_4_ titrations in a 33 mL solution (note the log *x*-axis). c–f) Profiles for 33 mL experiments were scaled so that the maximum titrant volume dosed occurs at the limit of the *x* axis (linear scale). Profiles for two control (no biomolecule) experiments are superimposed on each graph in gray for comparison.

In the 330 mL experiments, the rate of titrant dosing was approximately constant, resulting in a linear relationship between the time and the volume of titrant dosed (Figure 2a). Such a profile is generated when aragonite precipitation has little effect on the surface area for subsequent aragonite growth during the titration.^18^ In these experiments, the aragonite precipitation rate is calculated from the rate of titrant addition (to replace the CO_3_^2–^ and Ca^2+^ consumed during CaCO_3_ formation) normalized to the surface area of the seed.^18^ We test for differences in the aragonite precipitation rate between treatments using one-way ANOVA followed by Tukey’s pairwise comparison.

In the 33 mL experiments, the rates of titrant dosing accelerated as the experiment progressed (Figure 2b). This occurs as precipitation increases the surface area for aragonite growth during the experiment.^18^ The seed surface area to solution volume ratio was the same in the 330 and 33 mL experiments. However, nucleation of minor amounts of CaCO_3_ on the glass pH sensor or the titrant dosing tubes could increase the surface area for aragonite growth in the 33 mL experiments while having a negligible effect in the 330 mL experiments. We plot the titration profiles for 33 mL control experiments and those with varying concentrations of Asp and Asp_4_ so that the profiles can be directly superimposed (Figure 2 c–f). These plots demonstrate that the titrant dosing profile does not vary in shape between treatments. This confirms that although the surface area for aragonite growth increases in 33 mL, it does so in a similar manner in all the treatments; i.e., although the aragonite precipitation rate is usually slower with biomolecules (Figure 2b), the surface area for aragonite growth appears to increase in a similar manner (albeit over a different time scale) between treatments. We infer that the surface area for aragonite growth is the same in all the 33 mL experiments, and we use the total time to dose 0.7 mL of titrant to estimate the aragonite precipitation rate, assuming that the mean dosing time of 3738 s observed in the control titrations reflects a precipitation rate of 2011 μmol m^2^ h^–1^ (as observed in a 330 mL experiment with the same seed) and scaling dosing time to precipitation rate in a linear manner. By this method, we estimate a mean aragonite precipitation rate of 1442 μmol m^2^ h^–1^ in the treatment with 1 mM Asp, which is in reasonable agreement with a previously reported rate (1106 μmol m^2^ h^–1^).^18^

### 2.2 Amino Acid Analysis

The [Asp] and [Gly] of the intracrystalline fraction of the aragonite precipitates were determined by reverse-phase HPLC with fluorescence detection, following the method of Hendy et al.^44^ (a modification of Kaufman and Manley).^45^ <20 mg of aragonite was accurately weighed into a plastic microcentrifuge tube, bleached using 50 μL of 12% NaOCl per mg for 48 h (to oxidize any surficial amino acids), then sequentially rinsed with 18.2 MΩ H_2_O and methanol, and dried overnight. Samples precipitated with peptides were run as both free and hydrolyzed samples to determine if amino acids were incorporated in the peptide form (and therefore detectable in hydrolyzed samples only) or as free amino acids (i.e., after hydrolysis of the peptide in seawater during the titration or during precipitation). Samples precipitated in the presence of free amino acids were typically not hydrolyzed before analysis, but demineralized in 2 M HCl (10 μL/mg) and spun to dryness in a centrifugal evaporator. To hydrolyze the peptide bonds in the samples, <10 mg was accurately weighed into a 2 mL sterile glass vial (Wheaton) and 20 μL/mg 7 M HCl was added. After a flush with nitrogen, the vials were heated at 110 °C for 24 h. Upon removal, samples were dried in a centrifugal evaporator overnight. Both the free and hydrolyzed samples were rehydrated in a solution containing 0.01 mM l-homoarginine (as an internal standard) and analyzed using an Agilent 1100 HPLC with a fluorescence detector. 2 μL of sample was injected and mixed for 13 cycles with 2.2 μL of a derivatizing reagent (260 mM *n*-iso-l-butyryl l-cysteine (IBLC) and 170 mM *o*-phthaldialdehyde (OPA) in 1 M potassium borate buffer). The amino acids were separated on a C18 HyperSil BDS column (3 × 250 mm) at 25 °C using a gradient elution of three solvents (Table S1: sodium acetate buffer (solvent A; 23 mM sodium acetate trihydrate, 1.5 mM sodium azide, 1.3 μM EDTA, adjusted to pH 6.00 ± 0.01 with 10% acetic acid and 10 M sodium hydroxide), methanol (solvent C), and acetonitrile (solvent D)), at an initial flow rate of 0.56 mL/min increasing to 0.6 mL/min, and a 95 min cutoff. The final [amino acid] of the aragonite precipitated in vitro was calculated by correcting the [amino acid] of the final sample for the [amino acid] of the seed.

### 2.3 Raman Spectroscopy

Raman spectra were collected from the starting seeds and at least two replicate titrations of each treatment, except for the Asp_4_ treatment, which had no replicate. Spectra were collected using a Renishaw In-Via Qontor Raman microscope using a NIR 300 mW 785 nm solid-state laser set at 5% full power with a 1200 1/mm grating at a spectral resolution of 1 cm^–1^. The instrument was calibrated using the 520 cm^–1^ vibrational model of a Si standard. For each analysis (spectrum), the laser was focused onto the edge of particles, and the spectrum was collected between 100 and 1311 cm^–1^ for 2 s in each acquisition, with the cosmic ray removal function enabled, and 10 acquisitions were summed to give a final spectrum. Spectra were collected from 10 to 25 particles in each aragonite sample. The full width at half-maximum (fwhm) of the ν_1_ peak was estimated by fitting the ν_1_ peak between 1060 and 1120 cm^–1^ with a Voigt fit using the software Origin 2021 (OriginLab Corporation). Measured fwhm was corrected to true fwhm using the instrument spectral resolution.^46^

We also collected spectra for solid Gly, Asp, and Gly-Asp. These biomolecules exhibit multiple Raman bands between 120 and 1120 cm^–1^ (Figure S1), but the most intense bands for each biomolecule (i.e., at 893 cm^–1^ for Gly, at 940 cm^–1^ for Asp, and at 935 cm^–1^ for Gly-Asp) were not observed in the aragonite spectra, confirming that the biomolecule spectra do not affect the ν_1_-CO_3_ vibrational mode fwhm. The Raman spectrum of aqueous glycine is pH dependent^47^ but changes are minor and no Raman bands are observed at ∼1083 cm^–1^ that could interfere with the aragonite ν_1_ peak fwhm. Finally, we confirmed that the fwhm of the ν_1_ peak was not affected during analysis by collecting 12 spectra (120 acquisitions) on the same locations of particles precipitated with no biomolecule and with 1 mM Asp_4_ or 8.7 mM Asp (Figure S2).

We collected 50 spectra from three of the control precipitates and confirmed that the fwhm population was normally distributed (Shapiro–Wilk test, *p* = 0.93). We assume normal distributions for the other treatments, combine all fwhm data from replicates of the same treatment, and test for differences in the fwhm between treatments using one-way ANOVA followed by Tukey’s pairwise comparison.

### 2.4 Scanning Electron Microscopy

Scanning electron microscopy (SEM) images were collected for selected aragonite samples. Precipitates were mounted on aluminum pin stubs using double-sided carbon adhesive discs and viewed uncoated in a JEOL 7800F Prime (at the Nanocenter, University of York), using an accelerating voltage of 2.0 keV and a working distance of 10 mm.

## 3 Results

All data (precipitation rates, aragonite [amino acid], and Raman ν_1_ band fwhm) are included in Tables S2–S6. All precipitates exhibit Raman lattice mode peaks at ∼153 and 206 cm^–1^ and a dual peak (ν_4_) between 700 and 710 cm^–1^, indicating that all samples consist essentially of aragonite.^48,49^

### 3.1 Influence of Biomolecules on Aragonite Precipitation

**Figure 3:**
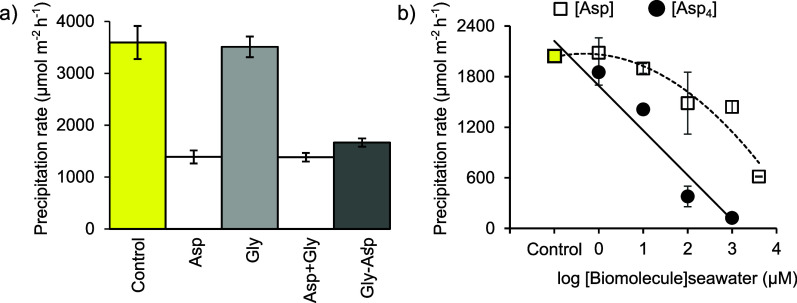
Aragonite precipitation rates in seawater at Ω_aragonite_ = 11 in a) 330 mL experiments with 2 mM biomolecules, where used, and b) 33 mL experiments at varying concentrations of Asp or Asp_4_. Lines indicate best-fit relationships between seawater [biomolecule] and the precipitation rate in b). Error bars are standard deviations of 5–7 replicates in a) and 2–3 replicates (except for 1000 μM Asp_4_, where *n* = 1) in b). In b), many error bars are smaller than the symbols.

Aragonite precipitation rate is significantly lower in the presence of 2 mM Asp, Asp+Gly, and Gly-Asp compared with the control (Figure 3 and Table 1). 2 mM Gly has no significant effect on the aragonite precipitation rate compared to the control, and neither Gly-Asp nor Asp+Gly results in a significant change to the aragonite precipitation rate compared to Asp in isolation.

**Table 1:** Summary of *p*-Values Generated in the ANOVA Analyses in this Study^a^^b^

ANOVA to Compare Aragonite Precipitation Rates between 330 mL Experiments (Figure 3a)				
	Asp	Gly	Gly-Asp	Gly + Asp
control	**3.5 × 10^–14^**	0.93	**7.9 × 10^–14^**	**3.3 × 10^–14^**
Asp	–	**3.8 × 10^–14^**	0.21	1.00
Gly	–	–	**1.6 × 10^–13^**	**3.4 × 10^–14^**
Gly-Asp	–	–	–	0.16

ANOVA to Compare Raman ν_1_ fwhm between 330 mL Experiments (Figure 4a)				
	Asp	Gly	Gly-Asp	Gly + Asp
control	**6.5 × 10^–12^**	0.35	**8.0 × 10^–10^**	**<1 × 10^–21^**
Asp	–	**3.6 × 10^–7^**	0.92	**0.0066**
Gly	–	–	**1.8 × 10^–5^**	**2.5 × 10^–14^**
Gly-Asp	–	–	–	**0.00021**

ANOVA to Compare Raman ν_1_ fwhm between 33 mL Experiments (Figure 4b)					
	1 μM Asp	10 μM Asp	100 μM Asp	1 mM Asp	4 mM Asp
control	0.97	**3.3 × 10^–5^**	**3.2 × 10^–9^**	**2.0 × 10^–3^**	**5.0 × 10^–6^**

	1 μM Asp_4_	10 μM Asp_4_	100 μM Asp_4_	1 mM Asp_4_
control	**8.4 × 10^–3^**	0.80	**1.7 x10**^**–5**^	**1.7 × 10^–5^**
4 mM Asp				**1.9 x10**^**–3**^

a We use one-way ANOVA and Tukey’s HSD to compare aragonite precipitation rates and Raman spectra ν1 FWHM between treatments with and without 2 mM biomolecules.

b *p*-values ≤0.05 are highlighted in bold.

Asp_4_ inhibits the aragonite precipitation rate at all the concentrations tested, with inhibition increasing from 9% at 1 μM to 94% at 1000 μM (Figure 3b). Asp marginally increases the aragonite precipitation rate at 1 μM and then progressively inhibits precipitation at higher concentrations. Asp_4_ slows aragonite precipitation to a much greater degree than Asp, even when the number of aspartic acid residues in solution is considered (Figure 3b).

### 3.2 Amino Acid Incorporation into Aragonite

[Asp] and [Gly] in aragonite precipitated with no added amino acids are both 0 pmol mg^–1^ in experiments analyzed for free amino acids and <212 and <156 pmol mg^–1^ respectively, for hydrolyzed samples (Table S4). These values are <2% of the maximum concentrations observed in the aragonite samples precipitated with biomolecules and are considered insignificant. In the case of aragonite precipitated with peptide, free amino acids contribute <2% of the total precipitate [amino acid] (Table S4), indicating that the amino acids detected in the aragonite are incorporated predominantly in their peptide form.

**Figure 4:**
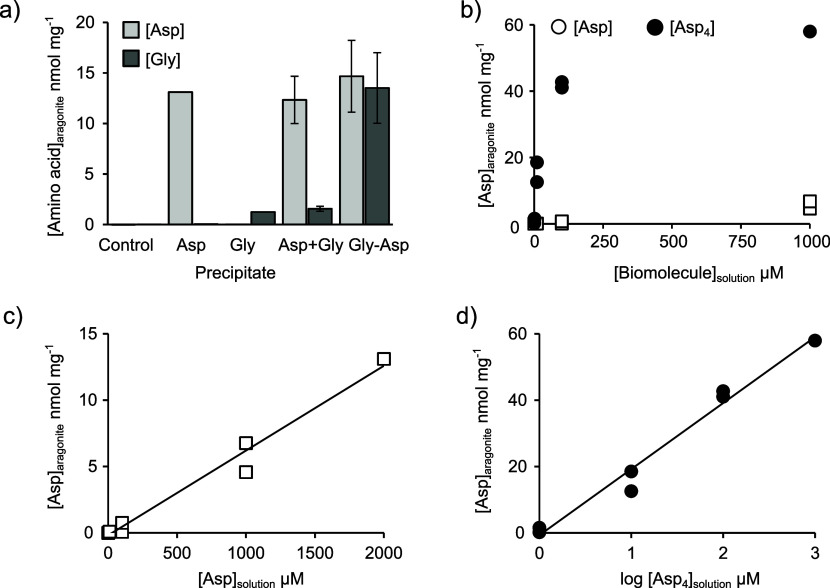
[Amino acid] in synthetic aragonite precipitated in vitro. Concentrations in a) aragonite precipitated in the presence of 2 mM of free amino acids, of the dipeptide Gly-Asp and in the presence of 2 mM Asp + 2 mM Gly (Asp+Gly) in combination, in the 330 mL experiments, and b) aragonite precipitated over variable concentrations of Asp and Asp_4_ in the 33 mL experiments. c) A linear plot for the Asp data including both 330 and 33 mL experiments, and d) a log plot for the Asp_4_ data. Seawater [biomolecule] indicates the concentration at the start of the experiment. In a), error bars indicate the standard deviation of duplicate analyses of repeat precipitations. In b–d, duplicate analyses are shown as separate points.

Asp and Gly are incorporated into aragonite precipitated in the presence of free forms of these amino acids (Figure 4). Asp is incorporated at more than 10 times the concentration of Gly from solutions of each amino acid at 2 mM, i.e., 13 nmol mg^–1^ for Asp versus 1.3 nmol mg^–1^ for Gly (Figure 4a). Both Asp and Gly are incorporated at similar concentrations in aragonite precipitated with the dipeptide Asp-Gly at 2 mM i.e., 14.7 nmol mg^–1^ for Asp versus 13.5 nmol mg^–1^ for Gly. When the two amino acids are simply put together in solution in their free forms (Asp+Gly), the amino acids are incorporated at similar concentrations to those observed when aragonite is precipitated with Asp or Gly only, i.e., Asp is preferentially incorporated into the precipitate (Figure 4a). Asp incorporation into aragonite increases as a function of seawater [Asp] and [Asp_4_] (Figure 4b). This relationship is linear in the experiments with [Asp] ranging from 0 to 2 mM (the maximum in our experiments; Figure 4c). Incorporation of Asp plateaus at higher concentrations in the Asp_4_ experiments (Figure 4b), and incorporation in these experiments is best fit with a plot of log[Asp]_solution_ versus [Asp]_aragonite_ (Figure 4d).

32 and 315 mg of aragonite were precipitated in the 33 and 330 mL experiments, respectively. We calculate the seawater [biomolecule] at the end of the precipitations, assuming that any biomolecule not incorporated in the aragonite remains in solution and that the incorporation of 1 nmol Asp_4_ increases aragonite [Asp] by 4 nmol (reflecting the number of residues in the peptide). In the free Asp experiments, <1% of the dissolved Asp is incorporated into the aragonite. However, approximately one-third of the Asp_4_ dissolved into seawater at the start of the titrations is incorporated into the aragonite at starting [seawater Asp_4_] of 1 and 10 μM, and about 10% is incorporated at [seawater Asp_4_] of 100 μM.

### 3.3 Biomolecules and Raman Aragonite Spectrum ν_1_ Fwhm

**Figure 5:**
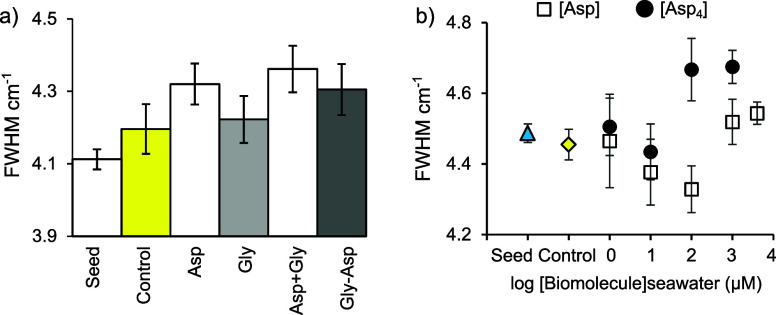
Raman ν_1_ fwhm in synthetic aragonite in a) the 330 mL experiments with 2 mM amino acids and peptides and b) the 33 mL experiments with varying concentrations of Asp and Asp_4_. 12–15 spectra were collected for each precipitate, and at least two precipitates (from replicate titrations) were analyzed in each treatment (except for 1 mM Asp_4_, where only one precipitate was produced). Data from multiple replicate titrations were combined for analysis. Bars/points represent means (*n* = 16–50) and error bars show 1 standard deviation.

The Raman ν_1_ fwhm increases significantly in synthetic aragonite precipitated with 2 mM Asp, 2 mM Gly-Asp, and 2 mM Asp + 2 mM Gly compared to the control (Table 1 and Figure 5a). 2 mM Gly does not significantly affect the fwhm. The aragonite precipitated with 2 mM Asp +2 mM Gly has a significantly broader fwhm than aragonite precipitated with 2 mM Asp only (Table 1).

The ν_1_ fwhm decreases significantly with 10 and 100 μM Asp but increases significantly with 1 and 4 mM Asp compared to the control (Table 1 and Figure 5b). Asp_4_ significantly increases the fwhm at 1, 100, and 1000 μM, but not at 10 μM (Table 1). The fwhm is significantly broader with 1 mM Asp_4_ compared to 4 mM Asp (Figure 5b and Table 1).

**Figure 6:**
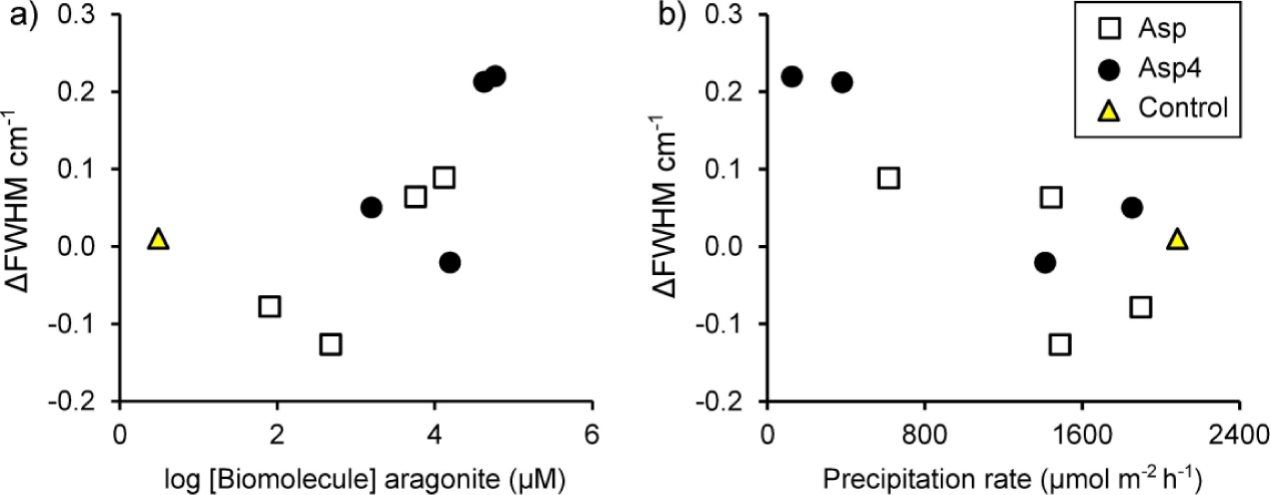
Δfwhm (deviation of fwhm in the sample from that of the control) as a function of a) seawater biomolecule concentration, b) aragonite amino acid concentration, and c) aragonite precipitation rate.

We plot the change in fwhm (Δfwhm i.e., the increase/decrease in fwhm compared to the relevant control) as a function of aragonite [Asp] (Figure 6a) and the aragonite precipitation rate (Figure 6b). Δfwhm is significantly related to aragonite precipitation in the Asp_4_ data set and when both Asp and Asp_4_ precipitations are combined, but all other relationships between Δfwhm and seawater or aragonite biomolecule concentrations or the precipitation rate are insignificant (Table 2).

**Table 2:** Coefficients of Determination (*r*^2^) and *p*-Values of Regression Analysis of Relationships between the ΔFWHM and Biomolecule Concentration or Aragonite Precipitation Rate^a^

	Δfwhm vs seawater [biomolecule]		Δfwhm vs aragonite [biomolecule]		Δfwhm vs precipitation rate	
	*r*^2^	*p*	*r*^2^	*p*	*r*^2^	*p*
Asp (*n* = 6)	0.11	0.52	0.11	0.51	0.20	0.37
Asp_4_ (*n* = 5)	0.67	0.088	0.39	0.26	0.82	**0.033**
all experiments combined (*n* = 10)	0.17	0.24	0.35	0.069	0.60	8.7 × 10^–3^

a *p-*values ≤0.05 are highlighted in bold.

### 3.4 Aragonite Morphology

**Figure 7:**
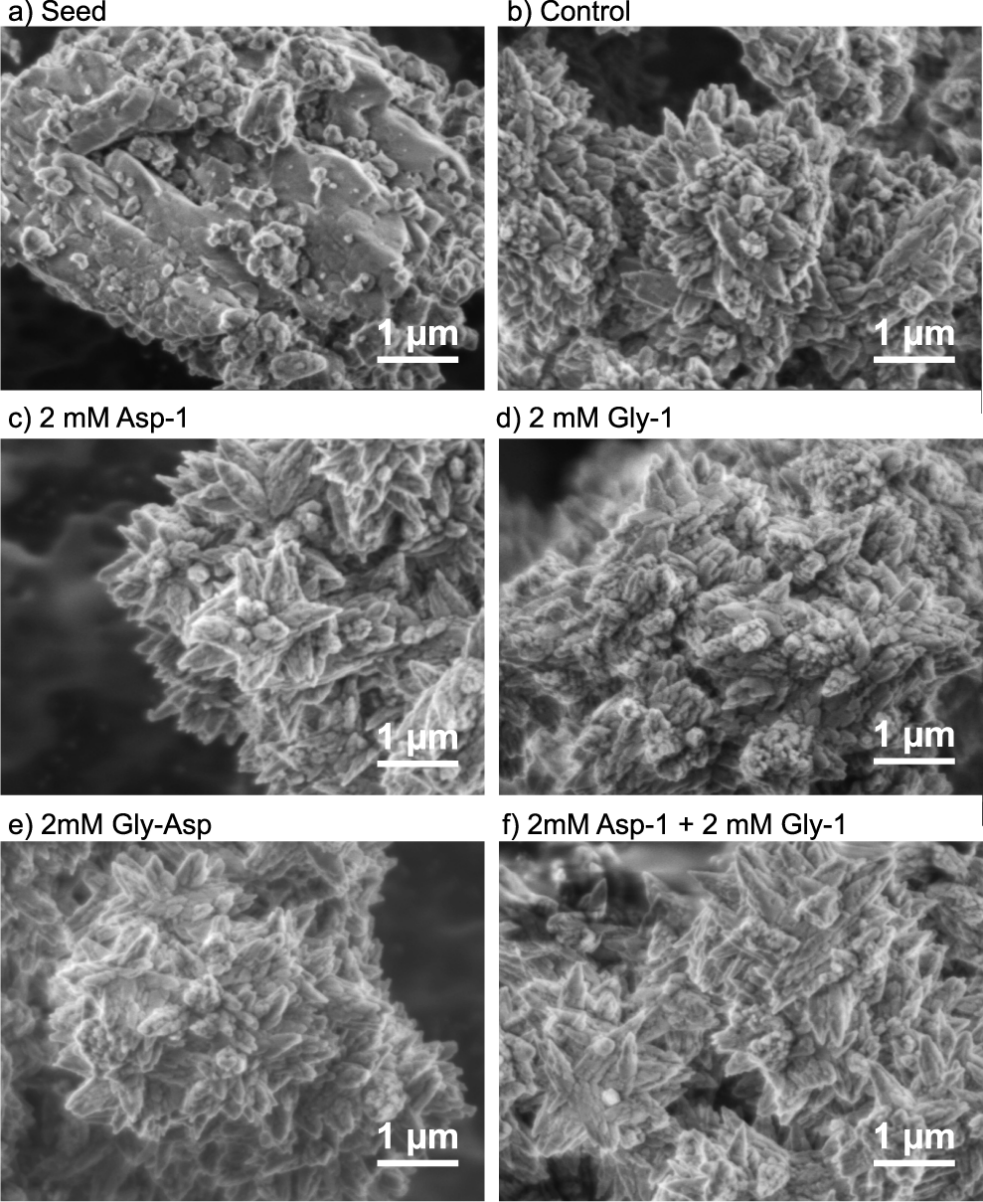
Scanning electron microscopy images of a) the coral seed and of aragonite precipitated in the 330 mL experiments: b) without biomolecule, c) with 2 mM Asp, d) with 2 mM Gly, e) with 2 mM Gly-Asp, and f) 2 mM Asp +2 mM Gly.

**Figure 8:**
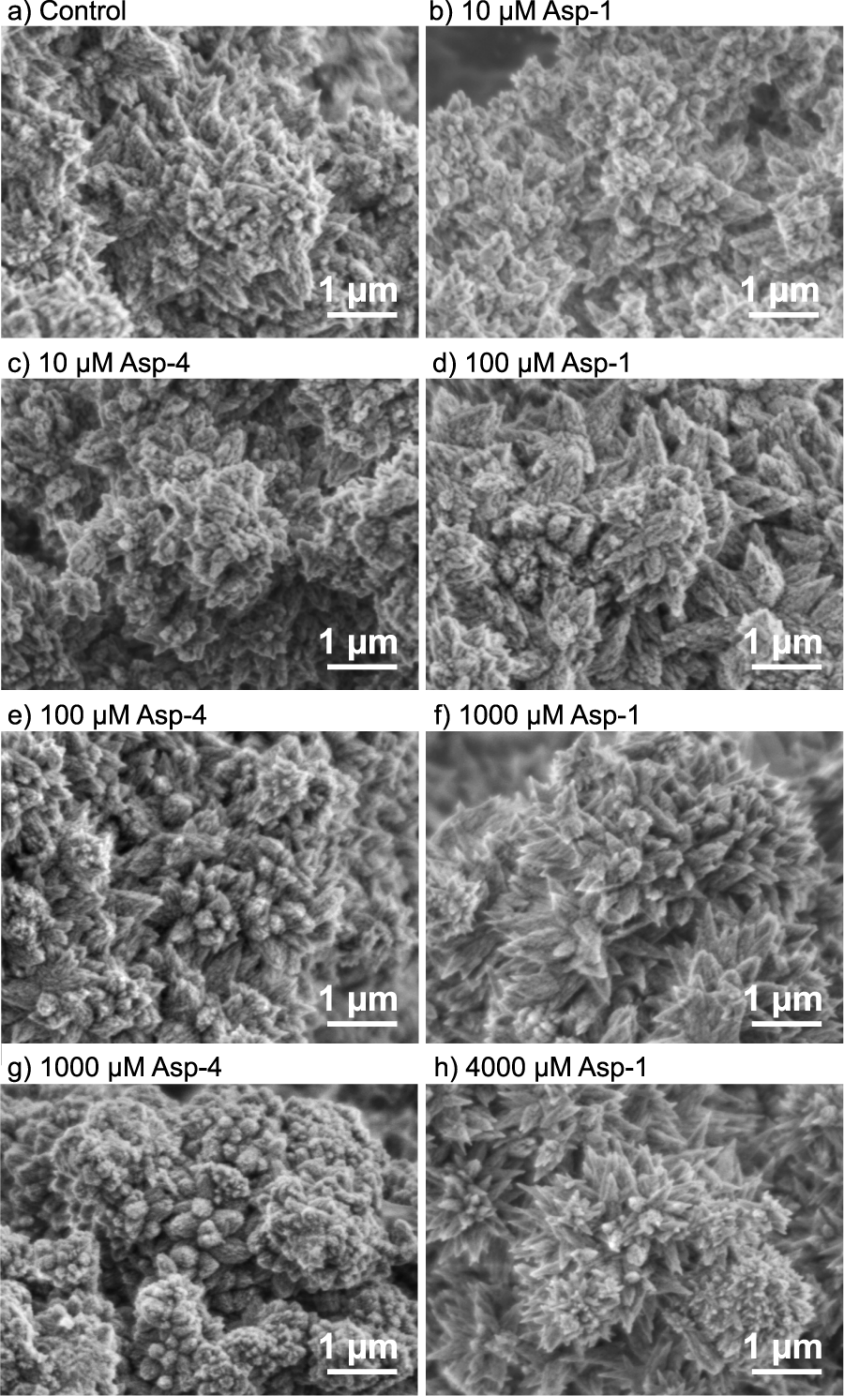
Scanning electron microscopy images of aragonite precipitated in the 33 mL experiments with a) no biomolecule, b), d), f), and h) with 10, 100, 1000, and 4000 μM Asp respectively, and c), e), and g) with 10, 100, and 1000 μM Asp_4_, respectively.

In all of the aragonite precipitated in the 330 mL experiments, the surface morphology is dominated by pyramidal clusters with crystals radiating in different directions from a common point (Figure 7). Differences in morphology between treatments are subtle, but aragonite crystals produced in the presence of 2 mM Gly are blunter than those observed in the controls (Figure 7b and d), while aragonite crystals produced with 2 mM Asp, Gly-Asp, and 2 mM Asp +2 mM Gly are spikier, with narrower tips than in the control (Figure 7c,e, and f). Similar clusters of aragonite crystals are observed in the 33 mL experiments (Figure 8), but in this case, the effects of the biomolecules are more pronounced. High concentrations of Asp (1000 and 4000 μM) generate pointier, spikier crystals than in the control (Figure 8a, f and h), while 100 and 1000 μM Asp_4_ results in blunt, rounder crystals (Figure 8e,g). Lower magnification images of all precipitates are included in the Figures S4 and S5.

## 4 Discussion

### 4.1 Biomolecules and Aragonite Precipitation

In the aragonite precipitated in the presence of peptides (Asp_4_ or Gly-Asp), the amino acids are predominantly (>98%) incorporated as peptides, as opposed to free amino acids, and for the purposes of this discussion, we assume that both peptides are stable and not hydrolyzed during the experiments. The observations in the two series of experiments show that peptides and amino acids can influence aragonite formation in different ways. Both Asp and Asp_4_ inhibit aragonite precipitation, but building aspartic acid into a tetra-peptide increases the inhibition of aragonite precipitation relative to the number of amino acid residues present; for example, the 1 mM Asp_4_ and the 4 mM Asp treatments contain the same number of aspartic acid residues in solution, yet the aragonite precipitation rate in the Asp_4_ treatment is ∼20% of that in the Asp treatment (Figure 3b). Similar effects are reported for Asp monomers and polymers in calcite.^16,17,50,51^ However, the Gly-Asp dipeptide has no greater effect on aragonite precipitation inhibition compared to Asp (Figure 3a and Table 1), indicating that the increase in molecule size in this case has no significant effect.

Amino acids and peptides affect CaCO_3_ formation by binding the aqueous ions required for mineral formation,^52^ by influencing the attachment of ions to an existing crystal surface,^7^ or by affecting the formation of CaCO_3_ precursor phases.^12^ Little CaCO_3_ formation is observed in unseeded experiments under the conditions of the present study, indicating that precipitation proceeds in the presence of an existing aragonite surface, i.e., the seed.^18^ In the present study, we observe incorporation of the amino acids and peptides into the intracrystalline fraction of the aragonite, indicating an interaction with the solid or a precursor phase. At pH 8.4, both Asp and Gly have a protonated amine group, NH_3_^+^, and a deprotonated α-carboxylate group, COO^–^.^31^ In calcite, deprotonated amino acid carboxyl groups (COO^–^) may attract to Ca^2+^ sites while protonated amine groups (NH_3_^+^) may interact with CO_3_^2–^ sites.^17^ In addition, the acidic side chain(s) of Asp (and we assume of Asp_4_) are deprotonated at physiological pH and above.^53^ This implies that all of the biomolecules used in this study can develop electrostatic interactions with the existing crystal surface. Once associated with the crystal surface, additives can inhibit CaCO_3_ growth by blocking ion attachment sites,^54,55^ may become entrapped in the crystal by progressive CaCO_3_ growth around the additive^55,56^ or may remain at the crystal surface and not be incorporated as the CaCO_3_ grows.^55^

The aragonite [Asp] and [Gly] in the present study are much lower than reported in previous studies of calcite^57,58^ where [Asp]_calcite_ and [Gly]_calcite_ reached up to ∼4 mol % and ∼7 mol %, respectively (equivalent to ∼400 nmol mg^–1^ and ∼700 nmol mg^–1^, respectively). [Amino acid]_calcite_ is positively related to [amino acid]_solution_,^58^ and in these previous studies, calcite was precipitated at higher [amino acid]_solution_ than used in the present study.^57,58^ In addition, [Asp]_calcite_ and [Gly]_calcite_ vary considerably between precipitations with comparable [amino acid]_solution_ in these previous studies, highlighting the importance of other experimental conditions in amino acid incorporation.

In our study, Asp_4_ is incorporated more effectively than Asp at comparable solution concentrations (Figure 4b), even assuming that each Asp_4_ molecule contributes 4 aspartic acid residues to the aragonite [Asp]. Asp is incorporated more effectively than Gly (Figure 4a). This likely reflects the strength of interactions between the biomolecule and the growing crystal surface. At the study pH, Asp_4_ has a higher negative charge (−4) than Asp (−1), which is itself more charged than the uncharged but polar Gly. Similarly, in calcite, both Asp and Gly and their monopeptides adsorb to the mineral surface, and Asp polymers adsorb more strongly than Asp.^17^

We precipitate aragonite over variable concentrations of Asp and Asp_4_, and we observe different relationships between seawater and aragonite concentrations in each case. In the Asp experiments, incorporation of Asp in the aragonite had little effect on seawater [Asp] (<1%) and aragonite [Asp] is proportional to seawater [Asp] over the entire analyzed range (1–2000 μM, Figure 4c). This suggests that adsorption sites of the amino acid onto the aragonite are not saturated during the precipitation. In contrast, in the Asp_4_ experiments, aragonite [Asp] flattens out at high seawater [Asp_4_] (Figure 4b), indicating that adsorption sites are becoming saturated as seawater [Asp_4_] increases. We estimate that the incorporation of Asp_4_ into the aragonite reduces the seawater [Asp_4_] by ∼10% at the highest seawater Asp_4_ and we consider this to be a small reduction. In the highest [Asp_4_] tested, aragonite [Asp] reaches 58 nmol mg^–1^, indicating that Asp_4_ and CaCO_3_ occur in the solid phase in a molar ratio of 1:690 (assuming that the incorporation of 1 nmol of Asp_4_ increases aragonite [Asp] by 4 nmol and that CaCO_3_ has a molecular mass of 100 g). In this experiment, the aragonite precipitation rate is reduced by >93%, suggesting that the incorporation of the biomolecule in this ratio severely suppresses crystal growth.

**Figure 9:**
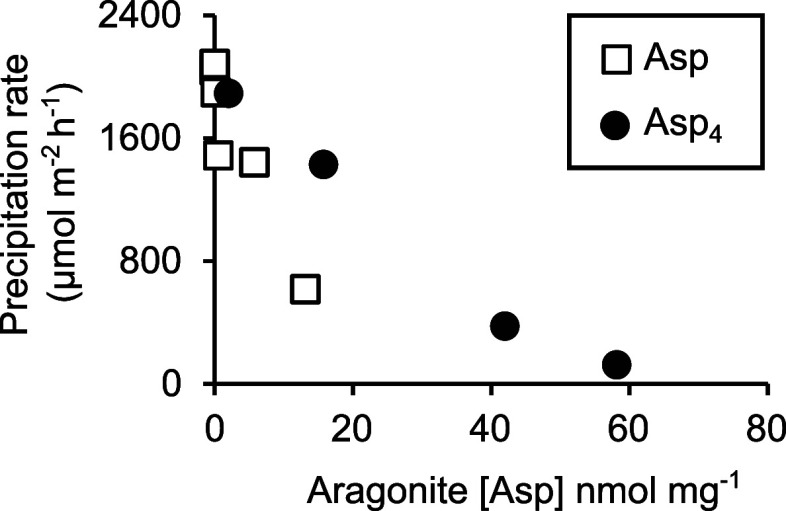
Aragonite precipitation rate as a function of aragonite [Asp] in the precipitations in the presence of Asp and Asp_4_.

To explore the relationship between biomolecule incorporation in aragonite and growth rate, we plot the aragonite precipitation rate as a function of aragonite [Asp] for the experiments conducted over varying seawater [Asp] and [Asp_4_] (Figure 9). Both Asp_4_ and Asp show significant inverse relationships between aragonite [Asp] and precipitation rate (*p* = 3.8 × 10^–5^ and 0.038 respectively), but the coefficient of determination (*r*^2^) is much higher for Asp_4_ (0.97) than for Asp (0.61). This is potentially because the low [Asp] tested here has little effect on the aragonite precipitation rate or on aragonite Asp incorporation. However, it is notable that similar aragonite precipitation rates (∼1400–1500 μmol m^–2^ h^–1^) are associated with aragonite [Asp] of <7 nmol mg^–1^ in aragonite precipitated in the presence of Asp, but with aragonite [Asp] of <12 nmol mg^–1^ in aragonite precipitated with Asp_4_. Put another way, our study indicates that each aspartic acid residue incorporated into the aragonite suppresses aragonite precipitation more in the free amino acid than in the peptide form. Potentially, this reflects the number of ion attachment sites that are blocked by each molecule, e.g., four free aspartic acid residues can potentially block 4 sites while a single Asp_4_ molecule may only be able to block 1 site. To summarize, the comparison of Asp and Asp_4_ indicates that Asp_4_ inhibits aragonite precipitation more than Asp at comparable solution concentrations (Figure 3) but that Asp reduces aragonite precipitation more effectively than Asp_4_ for each Asp residue incorporated into the aragonite (Figure 9).

Asp has markedly different effects on aragonite morphology compared to those of Asp_4_. In the presence of Asp, the pyramidal aragonite crystals become pointier (Figure 8h), while in the presence of Asp_4_, they become shorter and stubbier (Figure 8g). This indicates that the two biomolecules interact with the aragonite growth surface in different ways. Additives may alter calcite crystal morphology by adsorbing preferentially to particular faces,^59^ thereby promoting the development of other faces where the adsorbent is absent,^50,60,61^

In the present study, both the Gly-Asp dipeptide and free Asp have similar effects on the aragonite precipitation rate (Figure 3) and result in similar aragonite [Asp] (Figure 4a). Aragonite precipitated with this dipeptide has higher [Gly] compared to aragonite precipitated with only Gly or with both Asp and Gly in free amino acid form. This suggests that the incorporation of the dipeptide in aragonite is driven principally by the interaction of the Asp residue with the crystal surface and that the Gly residue has no discernible role, either in terms of its own chemistry or because it increases the size of the biomolecule. Gly has little impact on the aragonite precipitation rate when included as a single amino acid in isolation (Figure 3a).

Low concentrations of amino acids and peptides promote CaCO_3_ growth in calcite,^16,17,50^ For crystal growth from aqueous Ca^2+^ and CO_3_^2–^, the rate-limiting step is the desolvation of Ca^2+^ prior to binding to the crystal surface.^62^ Low concentrations of amino acids and peptides promote desolvation and decrease the energy barrier to the attachment of ions at the crystal surface.^16^ Low concentrations of aspartic acid (1 and 10 μM) accelerated aragonite precipitation in artificial seawater^18^ but this effect is less clear in the present study (Figure 3b). Here, varying [Asp] was tested in small volume experiments (33 mL) for direct comparison with the Asp_4_ data. We observe large variations between replicate titrations in these small volume experiments (coefficient of variation of up to 32%, Figure 3b) compared to the large volume experiments undertaken by Kellock et al.^18^ (coefficient of variation < 9%). This may reflect heterogeneity in the surface area of the seed used in the experiments. Seeds were produced by wet grinding a coral skeleton^18^ or a synthetic aragonite (33 mL experiments in the present study), resulting in irregular particles (e.g., Figure 7a). Variations in seed surface area will have more obvious effects on aragonite precipitation rate in the small volume experiments, which used a smaller seed mass (20 mg) compared to the large volume analogues (200 mg). Similarly, 2 mM Gly significantly reduced aragonite precipitation rate in a previous study in our laboratory^63^ but the effect was small (∼16% at pH_NBS_ 8.445 and Ω_aragonite_ ≈ 12.5), and this effect is not observed in the present study. The coefficient of variation of aragonite precipitation rates in replicate precipitations is up to 10% in the 330 mL experiments in the present study, so a small reduction or increase in precipitation rate is difficult to resolve without a larger number of experiments.^63^

Aragonite precipitation rates in the control experiments conducted at Ω = 11 varied from ∼3500 μmol h^–1^ m^–2^ in the experiments using the coral skeleton as a seed to ∼2040 μmol h^–1^ m^–2^ in the experiments using the synthetic aragonite seed (Figure 3). The origin of this difference is unclear. Potentially, the biomolecule content of the coral skeleton seed alters nucleation and accelerates aragonite precipitation.

### 4.2 Biomolecules and Aragonite Structure

High concentrations of all biomolecules, except glycine, increased the Raman spectrum ν_1_ band fwhm, interpreted as indicative of CO_3_ rotational disorder^33^ (Figure 5 and Table 1). All of the amino acids and peptides tested here are incorporated into the aragonite structure. Unit cell volume and Raman ν_1_ band fwhm were positively correlated across synthetic and biogenic aragonites^64^ and the inclusion of Asp and Gly expanded the *a* and *c* lattice parameters of calcite precipitated at ambient laboratory temperature,^57,58^ although the incorporation of Asp decreased the a lattice parameter in calcite precipitated at 134 °C.^65^ Lattice distortions are also observed in mollusk aragonite^66^ and calcite^67^ and in coral aragonite^64^ compared to geological minerals. The origin of these effects is not clear. Aspartic acid molecules have dimensions of 4–7 Å based on the bond length and angle^68^ while the unit cell of aragonite is ∼5 × 8 x 6 Å.^69^ Adsorbed molecules can become entrapped in the mineral lattice if they are buried in the crystal structure by subsequent mineral growth before desorption occurs.^55,56^ The biomolecules entrapped in aragonite in the present study may modify the local environment around the CO_3_ groups, thereby creating the disorder. We do not observe significant relationships between aragonite [Asp] and the shift in the aragonite fwhm from that observed in the control (Δfwhm, Figure 6a and Table 2). However, the numbers of analyses are small and little Asp is incorporated into aragonite at low [Asp], so it is difficult to resolve a relationship. We do observe a significant inverse relationship between the change in the fwhm of precipitates with Asp and Asp_4_ compared to that of the controls and aragonite precipitation rate for all of our synthetic precipitates (Figure 6b), indicating that there is a link between growth rate and aragonite crystallinity.

Besides biomolecule incorporation, CO_3_ rotational disorder may also reflect distortion of the CaCO_3_ lattice structure dependent on the incorporation of other impurity ions in the aragonite lattice.^64^ CaCO_3_ biominerals are impure and contain a variety of cations (e.g., Sr^2+^, Mg^2+^, Na^+^) and anions (e.g., B(OH)_4_^–^) which either substitute into the crystal lattice, e.g., Sr^2+^ replaces Ca^2+^ in aragonite^36^ or are incorporated by other means, as for Mg^2+^ in aragonite.^70^ Substitution of impurity ions causes dilation or contraction of the site and creates strain in the lattice.^71^ Fast mineral growth rates may enhance impurity ion incorporation if the impurities attach to the crystal surface and then become entrapped by rapid precipitation before they can detach,^72^ and indeed, the Raman spectrum ν_1_ fwhm has been used as a metric of Mg/Ca in calcite.^73^ The inverse relationship between aragonite precipitation rate and Raman spectrum ν_1_ band fwhm observed in the present study is contrary to a previous report where increased disorder was observed in aragonite precipitated with no additives at higher Ω_aragonite_, when mineral precipitation rates were rapid.^48^ Further work is required to identify how biomolecules and fluid Ω interact with mineral precipitation rate and impurity inclusion to affect the aragonite structure. Our observation that Gly-Asp has no greater effect on aragonite precipitation or ν_1_ peak fwhm than free aspartic acid, suggests that molecules of this size have limited effects on mineral growth rate or structure.

The Raman ν_1_ band fwhm is significantly narrower in aragonite precipitated with 10 and 100 μM Asp than in the controls. Similar narrowing was not observed in aragonite precipitated under similar conditions in a prior study.^18^ The fwhm of the control precipitated in the prior study (∼4.23 cm^–1^) is considerably lower than that observed in the present study (4.45 cm^–1^). This suggests that the Raman signature is influenced by the different seed materials used in each study (coral in Kellock et al, 2022^18^ and synthetic aragonite in the present study). Narrowing of the fwhm at low [Asp] may not have been apparent in the prior study, as the fwhm of the aragonite produced in the presence of 10 and 100 μM Asp in the present study (Figure 5) appears to be close to the fwhm of the seed used in Kellock et al., 2022.^18^

### 4.3 Implications for Biomineralization

Aspartic acid is the predominant amino acid in many coral skeletons,^1,23^ is abundant in mollusk shells^27−29^ and occurs in consecutive positions of coral acid-rich proteins, CARPs.^30^ It is unclear precisely how proteins influence skeletal formation;^23,25,74^ however, our study demonstrates that amino acids and peptides are incorporated into the aragonite. [Asp] in coral skeletons is measured as aspartic acid and asparagine combined (Asx) but is inferred to reflect predominantly Asp, as Asp:Arg is ∼10:1 in a coral acidic amino acid-rich skeletal protein.^30^ [Asx] and [Gly] in cultured coral skeletons range from ∼0.5 to 1.5 nmol mg^–1^ and 0.2 to 0.8 nmol mg^–1^ respectively.^23^ [Asx] and [Gly] in mollusk shells are up to 1 and 2 nmol mg^–1^ respectively.^75^ The [Asp] and [Gly] in the synthetic aragonites in the present study range from 0.07 to 58 nmol mg-1 (Figure 4c,d) and 1 to 16 nmol mg^–1^ (Figure 4a), respectively, indicating that the aragonites produced here have comparable amino acid contents to aragonitic biominerals.

Our study shows that low solution concentrations (1 μM) of Asp_4_ reduce aragonite precipitation, suggesting that CARPs may inhibit biomineralization. Direct pH and CO_3_^2–^ measurements of the coral extracellular calcification media suggest the media has Ω_aragonite_ of ≈12.^76^ At this Ω, homogeneous aragonite nucleation (in the absence of a nucleation surface) is not observed^77^ but precipitation is rapid onto an existing aragonite surface,^18,23,78^ as occurs when coral skeletal growth proceeds by ion-by-ion attachment onto the existing skeleton.^9^ CARPs may therefore act to inhibit aragonite precipitation, to prevent uncontrolled formation of the mineral phase, and to shape the coral skeleton.

## 5 Conclusion

All biomolecules are incorporated into aragonite to produce solids with [amino acids] comparable to those of biogenic aragonite. Asp, Asp_4_, and Asp-Gly inhibit aragonite precipitation at the solution concentrations required to generate these solids, suggesting that aspartic acid acts to suppress (rather than accelerate) biomineral formation. Biomolecule-driven changes in crystal morphology are very different between Asp and Asp4, perhaps indicating the preferential binding of each biomolecule to different crystal faces. Asp_4_ inhibits aragonite precipitation more than Asp at comparable solution concentrations, but Asp reduces aragonite precipitation more effectively than Asp_4_ for each Asp residue incorporated into the aragonite, potentially reflecting the ability of each biomolecule to block binding sites for precipitation.

## Supporting Information Available

Further details of the method to confirm minimal CO_2_ outgassing or invasion during titrations (the Method section and Figure S1); details of the HPLC solvent gradient (Table S1); all aragonite precipitation rates, aragonite [amino acid], and Raman ν_1_ band fwhm (Tables S2–S6); example Raman spectra of aragonite and biomolecules (Figure S2) and the fwhm of the Raman ν_1_ band in repeat analyses on the same location of aragonite with no biomolecule (control), Asp, and Asp_4_ (Figure S3); lower magnification scanning electron micrographs of all precipitates (Figures S4 and S5) (PDF)
