# Supplementary material for: Contrasting the Effects of Aspartic Acid and Glycine in Free Amino Acid and Peptide Forms on the Growth Rate, Morphology, Composition, and Structure of Synthetic Aragonites
Source: Cryst Growth Des. 2024 Nov 3;24(22):9379–90. doi: 10.1021/acs.cgd.4c00766 (PMC11583211; doi:10.1021/acs.cgd.4c00766)
Supplement: Supplementary file 1 — cg4c00766_si_001.pdf [file cg4c00766_si_001.pdf]

Supporting Information

Contrasting the effects of aspartic acid and glycine in free amino acid and peptide form on the growth rate, morphology and structure of synthetic aragonites

Giacomo Gardella, Maria Cristina Castillo Alvarez, Sam Presslee, Adrian A. Finch, Kirsty Penkman, Roland Kröger, Matthieu Clog & Nicola Allison

Method

We used the constant composition technique (Beck et al., 2013). The growth of $CaCO_3$ from solution decreased solution [DIC] and [total alkalinity] and thereby the pH of the experimental solution (see the $CaCO_3$ precipitation vector on Figure S1). The titration apparatus software detected this decrease in pH and added 0.45M Na_2CO_3 to the experimental solution to return the pH, [DIC] and [total alkalinity] to the original values (see the titrant dosing vector on Figure S1), although note that dosing essentially returned the experimental conditions to the value of the black dot. At the same time the software also added the same volume of a second titrant (0.4455 M $CaCl_2$ + 0.0045 M $SrCl_2$) to replace the Ca^{2+} used in $CaCO_3$ precipitation. Sr was replenished in the reaction vessel as it substitutes for Ca^{2+} ions in aragonite (Finch et al., 2003). Titrants were added automatically when the pH of the solution fell below 0.003 pH units of the set value. The standard deviation of pH measurements measured every 1 to 5 s during each titration was <0.005 pH units and we consider the pH of the solution to be essentially constant.

Experiments were conducted at conditions where the pCO_2 of the experimental solution was in equilibrium with the vessel headspace (~416 µatm) to avoid CO_2 invasion or outgas between the experimental solution and the headspace (see vectors in Figure S1). We measured the [dissolved inorganic carbon] of the experimental solutions at the start and end of a sub set of the experiments. The difference between [DIC] at the start and end of the precipitation was 4% on average and always <8%.This confirmed that CO_2 invasion or outgas was minimal and had little effect on the Ω of the experimental solution. The average change in [DIC] of 4% over a titration is equivalent to a change in $\Omega_{aragonite}$ of 0.4.

Figure S1. The impact of $CaCO_3$ precipitation, titrant dosing, CO_2 invasion and CO_2 outgas on the dissolved inorganic carbon (DIC) and total alkalinity and pH of the experimental solution. pH (NBS scale) contours are dropped onto the plot. The central black dot shows the composition of the solution at the start of each titration.

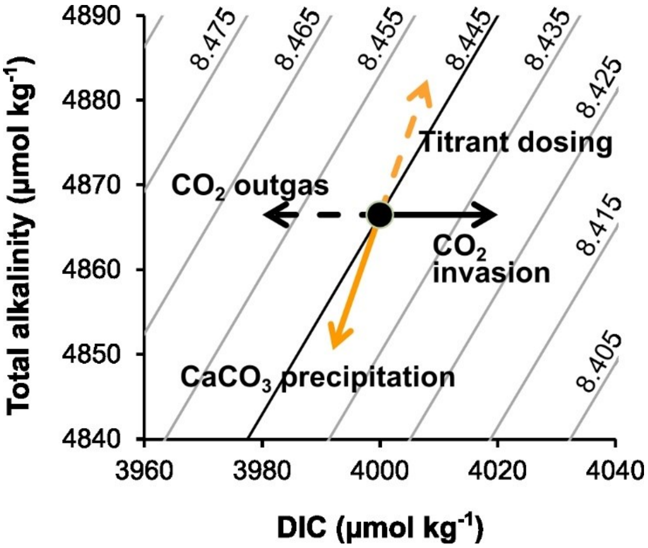

Figure S2. Raman spectra for aragonite (a control precipitation with no biomolecule), for glycine (Gly), for aspartic acid (Asp) and for the dipeptide (Gly-Asp).

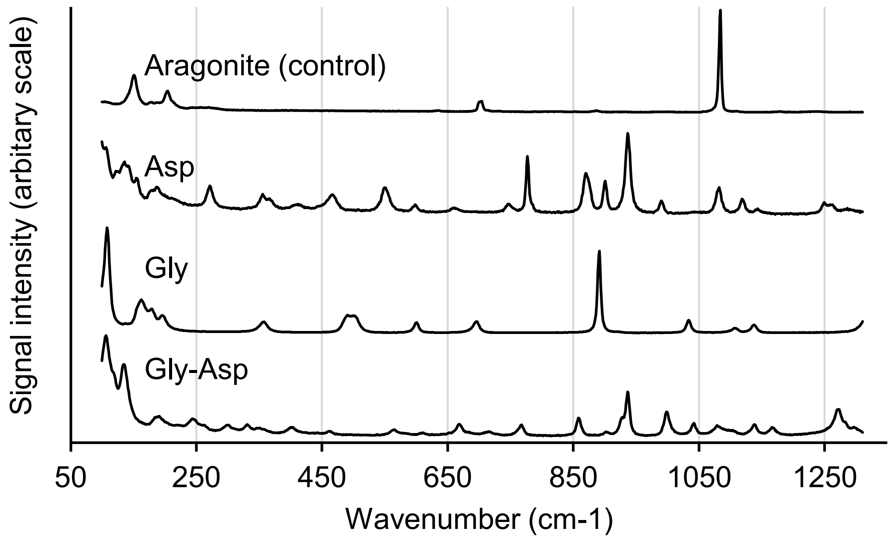

Figure S3. The FWHM of the υ_1 peak in the aragonite Raman spectrum for repeat analyses of the same location on particles of aragonites with no biomolecule (control), aspartic acid (Asp) and tetra-aspartic acid (Asp4).

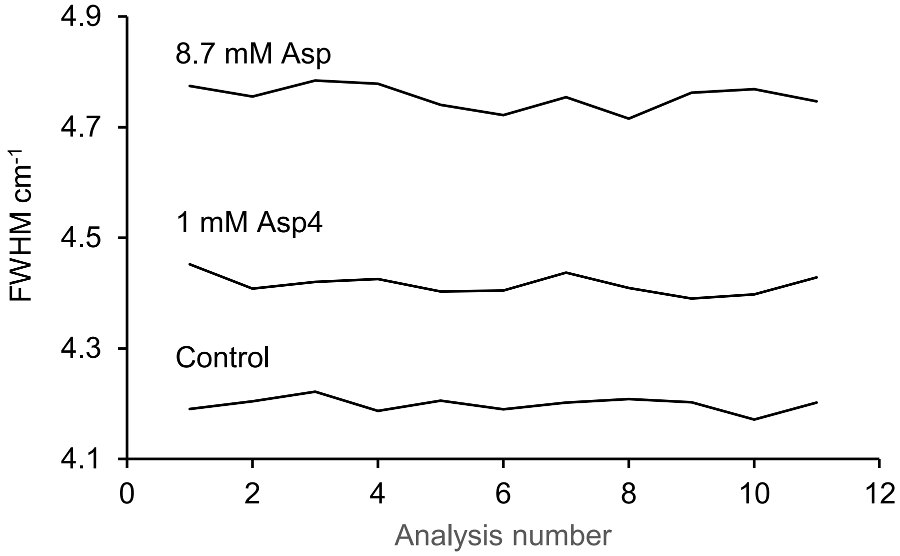

Figure S4. Scanning electron microscopy images of a) the coral seed, and of aragonite precipitated in the 330 mL experiments b) without biomolecule, c) with 2 mM Asp, d) with 2 mM Gly, e) with 2 mM Gly-Asp and f) 2 mM Asp + 2 mM Gly.

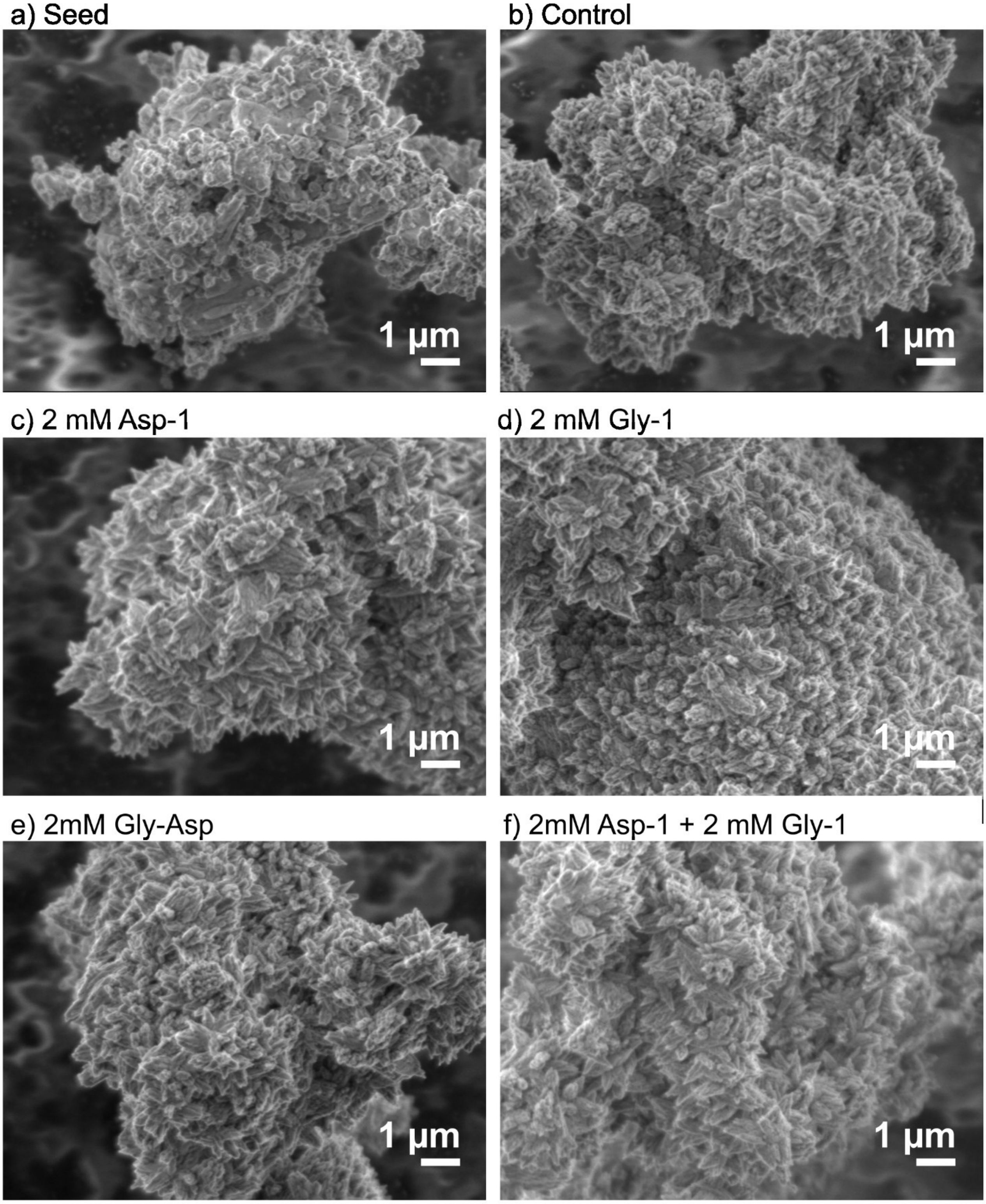

Figure S5. Scanning electron microscopy images of aragonite precipitated in the 33 mL experiments with a) no biomolecule, b), d), f) and h) with 10, 100, 1000 and 4000 μM Asp respectively and c), e) g) with 10, 100 and 1000 μM Asp_4 respectively.

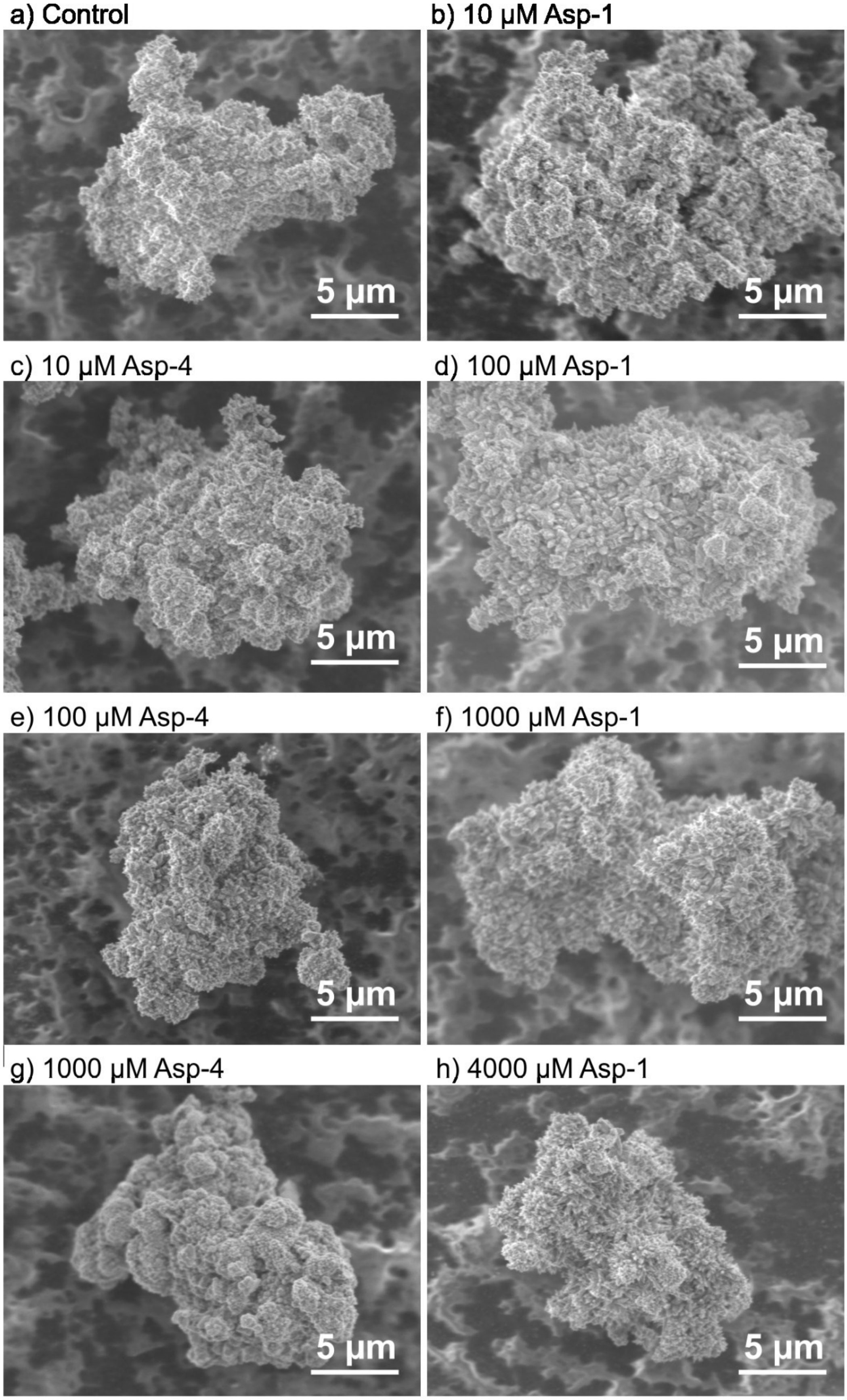

References

Beck, R.; Seiersten, M.; Andreassen, J. P. The constant composition method for crystallization of calcium carbonate at constant supersaturation. J. Cryst. Growth, 2013, 380, 187–196.

Finch, A. A.; Allison, N.; Sutton, S. R.; Newville, M. Strontium in coral aragonite: 1. Characterization of Sr coordination by extended absorption X-ray fine structure. *Geochim. Cosmochim. Acta*. **2003**, 67, 1197-202.

Contrasting the effects of aspartic acid and glycine in free amino acid and peptide form on the growth rate, morphology and structure of synthetic aragonites

Giacomo Gardella, Maria Cristina Castillo Alvarez, Sam Presslee, Adrian A. Finch, Kirsty Penkman, Roland Kröger, Matthieu Clog & Nicola Allison

Table S1. Reverse phase-HPLC gradient used for separation of chiral amino acids.

Time (min)	Solvent %			Flow rate (mL/min)
	A (Na acetate buffer)	C (MeOH)	D (ACN)	
0.0	95.0	5.0	0.0	0.56
31.0	76.6	23.0	0.4	0.56
95.0	46.2	48.8	5.0	0.60
95.9	0.0	95.0	5.0	0.60
99.0	0.0	95.0	5.0	0.60
100.0	95.0	5.0	0.0	0.60
115.0	95.0	5.0	0.0	0.56

Contrasting the effects of aspartic acid and glycine in free amino acid and peptide form on the growth rate, morphology and structure of synthetic aragonites

Giacomo Gardella, Maria Cristina Castillo Alvarez, Sam Presslee, Adrian A. Finch, Kirsty Penkman, Roland Kröger, Matthieu Clog & Nicola Allison

Table S2. Aragonite precipitation rates (µmol m-2 h-1) in 330 mL experiments with and without 2 mM of biomolecules.

Replicate	Control	Asp	Gly	Asp+Gly	Gly-Asp
1	3925	1588	3394	1471	1639
2	3122	1367	3211	1441	1586
3	3961	1313	3796	1234	1797
4	3461	1409	3715	1405	1638
6	3342	1258	3409	1370	1659
7	3497		3516	1367	
8	3829		3514		
Mean	3591	1387	3508	1381	1664
Standard deviation	319.635	126.057	198.503	82.839	79.4197

Contrasting the effects of aspartic acid and glycine in free amino acid and peptide form on the growth rate, morphology and structure of synthetic aragonites

Giacomo Gardella, Maria Cristina Castillo Alvarez, Sam Presslee, Adrian A. Finch, Kirsty Penkman, Roland Kröger, Matthieu Clog & Nicola Allison

Table S3. Aragonite precipitation rates (μmol m-2 h-1) in 33 mL experiments with and without biomolecules.

**Replicate**	1	2	3	Mean	Standard deviation
**Control**	2015	2082	2023	2040	36
**1 μM Asp**	2208	1958		2083	177
**10 μM Asp**	1855	1940		1897	60
**100 μM Asp**	1895	1380	1182	1486	368
**1000 μM Asp**	1389	1494		1442	75
**4000 μM Asp**	620	611		615	6
**1 μM Asp4**	1743	1964		1853	156
**10 μM Asp4**	1455	1367		1411	62
**100 μM Asp4**	294	465		380	121
**1000 μM Asp4**	125			125	

Contrasting the effects of aspartic acid and glycine in free amino acid and peptide form on the growth rate, morphology and structure of synthetic aragonites

Giacomo Gardella, Maria Cristina Castillo Alvarez, Sam Presslee, Adrian A. Finch, Kirsty Penkman, Roland Kröger, Matthieu Clog & Nicola Allison

Table S4. Aragonite [amino acid] in all seeds and precipitated aragonite (pmol/mg). Columns show [amino acid] measured in the collected solid (seed + precipitate) and estimated in the precipitate only after correcting for the composition of the starting seed.

	[Amino acid] in solid				[Amino acid] in precipitate only			
	Free [Asp]	Hydrolysed [Asp]	Free [Gly]	Hydrolysed [Gly]	Free [Asp]	Hydrolysed [Asp]	Free [Gly]	Hydrolysed [Gly]
Coral seed	13	1313	36	295				
Synthetic seed	0	9	1	43				
330 mL experiments using 2 mM biomolecules								
Control	5	616	16	210	0	173	3	156
Asp	8022	na	42	na	13107	na	46	na
Gly	14	477	781	1249	14	0	1254	1855
Asp-Gly 1	22	7944	0	6866	27	12154	0	11038
Asp-Gly 2	6	11018	5	9894	1	17180	0	15989
Asp+Gly 1	8560	16324	1082	2523	13986	25855	1746	3938
Asp+Gly 2	6542	na	867	na	10687	na	1395	na
33 mL experiments using [Asp] uM								
Control	2	134	18	119	3	212	28	167
1	2	na	17	na	3	na	27	na
10	53	na	17	na	86	na	27	na
10	45	na	16	na	73	na	25	na
100	471	na	0	na	765	na	0	na
1000	2824	3583	0	0	4589	5816	0	0
1000	4167	4260	0	0	6771	6917	0	0
33 mL experiments using [Asp4] uM								
Control	2	134	18	119	3	212	28	167
1	10	963	0	0	16	1559	0	0
10	82	11397	0	0	133	18514	0	0
10	53	7735	17	0	86	12564	27	0
100	286	26303	0	0	465	42737	0	0
100	378	25297	0	0	614	41102	0	0
1000	591	35669	0	0	960	57956	0	0

Contrasting the effects of aspartic acid and glycine in free amino acid and peptide form on the growth rate, morphology and structure of synthetic aragonites

Giacomo Gardella, Maria Cristina Castillo Alvarez, Sam Presslee, Adrian A. Finch, Kirsty Penkman, Roland Kröger, Matthieu Clog & Nicola Allison

Table S5. FWHM of υ1 peak in aragonite Raman spectrum in 330 mL experiments.

Sample FWHM cm-1

Replicate	Seed	Control - titration 1	Control - titration 2	Control - titration 3	Asp 2 mM - titration 1	Asp 2 mM - titration 2	Asp 2 mM + Gly 2 mM - titration 1	Asp 2 mM + Gly 2 mM - titration 2	Asp 2 mM + Gly 2 mM - titration 3	Gly-Asp 2 mM - titration 1	Gly-Asp 2 mM - titration 2	Gly 2 mM titration 1	Gly 2 mM titration 2	Gly 2 mM titration 3
1	4.145	4.165	4.181	4.121	4.262	4.225	4.448	4.428	4.316	4.277	4.252	4.202	4.401	4.216
2	4.131	4.119	4.263	4.253	4.377	4.383	4.424	4.469	4.344	4.365	4.210	4.257	4.183	4.313
3	4.176	4.124	4.306	4.199	4.411	4.429	4.409	4.429	4.378	4.437	4.374	4.263	4.324	4.288
4	4.089	4.271	4.143	4.188	4.421	4.318	4.527	4.344	4.457	4.357	4.366	4.215	4.326	4.209
5	4.168	4.189	4.392	4.251	4.305	4.295	4.404	4.407	4.466	4.281	4.341	4.190	4.157	4.238
6	4.153	4.275	4.309	4.261	4.351	4.360	4.402	4.473	4.418	4.412	4.367	4.327	4.246	4.210
7	4.127	4.196	4.239	4.338	4.373	4.281	4.323	4.443	4.277	4.194	4.368	4.219	4.299	4.222
8	4.132	4.215	4.136	4.153	4.316	4.341	4.422	4.221	4.377	4.173	4.372	4.342	4.268	4.130
9	4.116	4.250	4.269	4.235	4.305	4.257	4.357	4.445	4.466	4.355	4.296	4.318	4.110	4.196
10	4.102	4.242	4.202	4.187	4.279	4.340	4.387	4.400	4.437	4.299	4.246	4.206	4.192	4.258
11		4.200	4.290	4.126	4.426	4.351	4.334	4.321	4.383	4.305	4.343	4.323	4.178	4.220
12		4.194	4.130	4.184	4.381	4.336		4.416	4.435	4.315	4.332	4.234	4.192	
13		4.196	4.180		4.395				4.367	4.427	4.408		4.292	
14		4.253												
15		4.288												
16		4.230												
17		4.195												
18		4.326												
19		4.270												
20		4.319												
21		4.074												
22		4.223												
23		4.077												
24		4.219												
25		4.210												

Contrasting the effects of aspartic acid and glycine in free amino acid and peptide form on the growth rate, morphology and structure of synthetic aragonites

Giacomo Gardella, Maria Cristina Castillo Alvarez, Sam Presslee, Adrian A. Finch, Kirsty Penkman, Roland Kröger, Matthieu Clog & Nicola Allison

Table S6. FWHM of υ_1 peak in aragonite Raman spectrum in 33 mL experiments.

Sample FWHM cm-1

Replicate	Seed	Control - titration 1	Control - titration 2	Control - titration 3	1 µM Asp - titration 1	1 µM Asp - titration 2	10 µM Asp - titration 1	10 µM Asp - titration 2	100 µM Asp - titration 1	100 µM Asp - titration 2	1000 µM Asp - titration 1	1000 µM Asp - titration 2	4000 µM Asp - titration 1	4000 µM Asp - titration 2	1 µM Asp4 - titration 1	1 µM Asp4 - titration 2	10 µM Asp4 - titration 1	10 µM Asp4 - titration 2	100 µM Asp4 - titration 1	100 µM Asp4 - titration 2	1000 µM Asp4 - titration 1
1	4.440	4.430	4.497	4.486	4.386	4.730	4.337	4.274	4.549	4.288	4.501	4.628	4.604	4.592	4.617	4.582	4.580	4.516	4.656	4.600	4.763
2	4.493	4.432	4.506	4.474	4.534	4.631	4.411	4.321	4.371	4.292	4.435	4.592	4.573	4.553	4.422	4.450	4.559	4.370	4.435	4.751	4.742
3	4.533	4.434	4.509	4.408	4.390	4.724	4.368	4.420	4.390	4.332	4.448	4.528	4.547	4.558	4.496	4.529	4.547	4.475	4.632	4.796	4.683
4	4.538	4.389	4.479	4.447	4.441	4.423	4.429	4.394	4.422	4.359	4.419	4.561	4.524	4.581	4.553	4.540	4.519	4.273	4.742	4.806	4.647
5	4.516	4.486	4.418	4.512	4.368	4.612	4.392	4.294	4.430	4.363	4.560	4.668	4.578	4.542	4.540	4.475	4.522	4.430	4.760	4.664	4.701
6	4.504	4.404	4.477	4.504	4.463	4.649	4.453	4.294	4.386	4.354	4.504	4.605	4.586	4.596	4.556	4.471	4.510	4.366	4.647	4.774	4.690
7	4.483	4.449	4.483	4.523	4.226	4.613	4.569	4.267	4.288	4.291	4.492	4.611	4.564	4.544	4.657	4.418	4.476	4.452	4.533	4.685	4.734
8	4.505	4.479	4.458	4.500	4.437	4.364	4.498	4.302	4.389	4.283	4.547	4.550	4.599	4.569	4.700	4.396	4.482	4.419	4.765	4.675	4.772
9	4.514	4.463	4.579	4.484	4.481	4.477	4.455	4.362	4.403	4.261	4.487	4.531	4.590	4.572	4.487	4.534	4.534	4.362	4.667	4.629	4.630
10	4.496	4.509	4.562	4.444	4.393	4.615	4.573	4.255	4.413	4.379	4.436	4.495	4.563	4.559	4.535	4.485	4.474	4.330	4.644	4.774	4.656
11	4.533	4.421	4.490	4.513	4.387	4.502	4.512	4.348	4.309	4.288	4.524	4.563	4.547	4.591	4.620	4.465	4.421	4.377	4.674	4.689	4.618
12	4.490		4.550	4.462	4.244	4.576	4.478	4.330	4.350	4.271	4.484	4.598	4.603	4.536	4.638	4.435	4.524	4.441	4.788		4.711
13	4.528		4.471	4.500	4.406	4.545	4.542	4.462	4.338	4.277		4.634	4.568	4.474			4.489	4.363	4.687		4.658
14	4.529			4.509			4.455	4.331				4.547	4.590	4.574							4.733
15				4.452								4.481	4.540	4.485							4.700
16				4.412								4.543									4.665
17												4.592									
18												4.600									
